# Supplementary material for: Development of New Probe-Based Real-Time RT-qPCR Assays for the Detection of All Known Strains of Bovine Ephemeral Fever Viruses
Source: Viruses. 2025 Mar 13;17(3):407. doi: 10.3390/v17030407 (PMC11945983; doi:10.3390/v17030407)
Supplement: Supplementary file 1 [file viruses-17-00407-s001.zip › viruses-3452342-supplementary.pdf]

Table S1. Raw data on reproducible quality tests of extracted RNA from positive field samples.

		Assay type / RT-qPCR kit															
		Stram/Quanta		Erster/ Sensi		Quanta		Quanta		AgPath		AgPath		Clara		Clara	
Sample (replicate)	Source	Ct	ß-ACT	Ct	Melt T	MIX2	ß-ACT	MIX8	ß-ACT	MIX 2	ß-ACT	MIX 8	ß-ACT	MIX 2	ß-ACT	MIX 8	ß-ACT
A(1)	w.b.	NA	29.1	28.35	78.36	34.16	30.56	32.71	31.33	31.54	27.01	31.11	27.84	32.71	28.99	30.87	29.34
A(1)	w.b.	NA	28.84	26.96	78.36	33.92	30.28	32.56	31.17	31.01	27.76	30.62	28.28	32.43	29.15	30.75	29.42
A(2)	w.b.	NA	28.69	26.67	78.05	33.58	30.55	33.06	32.26	31.17	27.24	30.35	28.04	31.97	29.01	30.73	29.84
A(2)	w.b.	NA	28.56	26.97	78.05	33.54	30.4	32.15	30.85	31.08	27.49	30.39	27.76	31.96	28.79	30.73	29.42
A(3)	w.b.	NA	28.82	26.18	78.35	33.16	30.93	31.58	31.98	30.66	28.1	29.57	27.89	31.74	29.63	30.04	30.59
A(3)	w.b.	NA	29.22	25.77	78.35	32.9	31.05	31.68	32.07	30.35	28.13	29.5	28.15	31.78	30.16	30.18	30.97
B(1)	w.b.	NA	30.66	29.18	78.2	35.69	31.77	35.11	33.0	33.84	29.32	33.38	32.58	34.66	30.52	32.83	32.27
B(1)	w.b.	NA	30.32	29.79	78.35	35.84	32.23	34.54	32.72	33.19	28.56	32.95	29.7	35.73	30.79	32.46	31.48
B(2)	w.b.	NA	30.53	29.63	78.36	36.58	32.61	35.52	32.7	33.91	29.33	32.62	29.64	36.6	32.57	32.69	32.18
B(2)	w.b.	NA	30.1	30.35	78.36	36.93	31.6	35.79	32.28	32.99	28.82	33.32	29.93	35.43	31.42	32.55	32.3
B(3)	w.b.	NA	30.58	28.6	78.2	34.96	32.23	33.48	33.44	35.01	32.93	32.42	30.33	34.89	31.82	32.4	31.96
B(3)	w.b.	NA	30.57	28.8	78.2	35.21	32.12	33.99	33.37	32.93	30.13	32.22	30.19	34.18	31.65	31.7	31.51
C(1)	w.b.	25.86	26.58	15.34	80.02	21.74	28.87	19.93	29.06	19.74	26.12	18.41	26.15	20.15	27.56	18.81	28.38
C(1)	w.b.	26.34	26.41	15.22	80.02	22.41	26.68	19.63	28.79	19.67	25.46	18.53	26.01	19.81	27.61	18.69	28.56
C(2)	w.b.	26.22	26.34	15.56	80.02	22.0	28.44	20.2	28.46	20.03	26.26	18.78	26.02	20.02	27.04	18.83	27.72
C(2)	w.b.	26.39	26.63	15.58	79.87	22.11	28.72	20.13	28.79	19.97	26.29	18.81	26.12	20.09	26.93	19.22	27.93
C(3)	w.b.	25.49	26.61	13.86	79.72	20.56	28.15	18.18	27.17	18.44	25.29	16.31	25.75	18.93	26.6	17.8	27.25
C(3)	w.b.	25.38	26.23	14.44	79.87	20.48	28.43	17.97	27.52	18.74	25.8	17.65	26.01	19.1	26.81	17.56	27.64
D(1)	b.coat	NA	25.85	22.44	78.05	26.48	27.75	25.02	28.17	24.11	24.97	23.47	25.43	25.17	26.6	23.55	27.65
D(1)	b.coat	NA	26.61	21.92	78.05	26.66	27.89	24.4	28.71	23.8	24.58	22.92	24.92	24.88	26.66	22.66	27.04
D(2)	b.coat	NA	26.21	21.7	78.05	26.22	27.57	24.85	28.25	24.0	24.68	23.5	25.31	24.12	25.3	23.23	26.52
D(2)	b.coat	NA	25.16	22.31	78.05	26.4	27.41	24.96	28.62	23.91	24.53	26.18	29.05	24.43	25.55	23.22	26.11
D(3)	b.coat	NA	26.07	22.31	78.05	27.03	28.27	25.71	29.17	24.38	24.82	23.42	25.21	24.93	26.07	23.53	26.94
D(3)	b.coat	NA	26.05	22.23	78.05	27.03	28.28	25.57	28.73	24.27	24.66	23.52	24.67	24.96	26.44	23.34	26.89

Stram- assay designed by Stram et al. [21]; Erster- assay designed by Erster et al.[17]; Sensi- SensiFAST SYBR® No-ROX Kit (Bioline) was used for Erster et al. assay [17]; all the last- comparative tests of Mix-2 and Mix-8 recently validated assays; Ct- cycle threshold; w.b.- whole blood;b.coat- buffy coat; NA- not amplified; MeltT- melting temperature; ß -ACT- ß- actin; Quanta - qScript XLT One-Step RT-qPCR ToughMix(Quantabio); AgPath- AgPath-ID™ One-Step RT-PCR Kit (Life technologies); Clara- Clara™ Probe 1-Step Mix No-ROX (PCR biosystems); Promega- GoTaq 1-Step RT-qPCR System (Promega); Takara- One Step PrimeScript™ RT-PCR Kit (Takara Bio Inc.).

Table S2a. Raw data on evaluation tests of the limit of detection (LOD) based on Log10 dilution series in water of the BEFV strains.

		RT-qPCR kit and system																							
		Stram		Erster		Quanta		Quanta		AgPath		AgPath		Promega		Promega		TaKaRa		TaKaRa		Clara		Clara	
strain	source	Ct	b-ACT	Ct	Tm	MIX2	b-ACT	MIX8	b-ACT	MIX2	b-ACT	MIX8	b-ACT	MIX2	b-ACT	MIX8	b-ACT	MIX2	b-ACT	MIX8	b-ACT	MIX2	b-ACT	MIX8	b-ACT
AUS 10^{-2}	Vero	24.23	27.65	20.59	79.45	28.27	32.73	25.47	34.32	26.89	29.02	24.69	30.5	27.06	31.07	25.26	30.76	31.82	35.97	30.25	35.21	27.51	34.32	26.06	31.22
AUS 10^{-2}	Vero	23.21	27.51	20.45	79.45	28.64	33.89	24.79	33.77	27.61	31.97	25.2	30.87	27.53	30.74	25.61	31.48	32.02	36.28	30.84	34.65	27.43	33.39	26.08	34.69
AUS^{-3}	Vero	27.93	31.3	23.78	79.29	32.35	36.4	29.63	40.66	30.79	35.75	28.8	35.03	28.44	28.06	29.52	NA	38.1	NA	35.62	36.75	30.75	42.39	29.55	37.96
AUS^{-3}	Vero	27.16	31.46	23.86	79.29	32.13	38.5	29.28	NA	30.89	34.68	29.24	33.96	29.96	30.88	29.83	34.99	NA	34.99	35.91	NA	30.93	NA	29.51	37.66
AUS^{-4}	Vero	NA	NA	26.67	79.75	38.33	NA	37.38	NA	36.39	NA	35.09	NA	36.11	NA	36.92	NA	NA	NA	NA	NA	36.6	NA	36.19	NA
AUS^{-4}	Vero	32.85	37.14	27.71	79.6	38.32	NA	35.87	NA	NA	NA	33.95	NA	NA	NA	34.88	NA	NA	NA	NA	NA	35.9	NA	37.71	NA
AUS^{-5}	Vero	NA	NA	31.42	79.74	41.28	NA	38.18	NA	37.09	NA	NA	NA	37.99	NA	37.8	NA	NA	NA	NA	NA	NA	NA	NA	35.99
AUS^{-5}	Vero	NA	NA	31.55	79.29	38.84	NA	38.96	NA	NA	NA	37.48	NA	NA	NA	36.87	NA	NA	NA	NA	NA	38.23	NA	38.23	NA
AUS^{-6}	Vero	NA	NA	33.91	78.84	NA	NA	NA	NA	NA	NA	NA	NA	NA	NA	NA	NA	NA	NA	NA	NA	NA	NA	NA	NA
AUS^{-6}	Vero	NA	NA	NA	NA	NA	NA	NA	NA	38.76	NA	NA	NA	NA	NA	NA	NA	NA	NA	NA	NA	NA	NA	NA	NA
AUS^{-7}	Vero	NA	NA	NA	NA	NA	NA	NA	NA	NA	NA	NA	NA	NA	NA	NA	NA	NA	NA	NA	NA	NA	NA	NA	NA
AUS^{-7}	Vero	NA	NA	NA	NA	NA	NA	NA	NA	NA	NA	NA	NA	NA	NA	NA	NA	NA	NA	NA	NA	NA	NA	NA	NA
ISR-21^{-2}	BSR	NA	NA	20.41	78.08	27.31	35.11	26.12	34.3	27.15	32.38	25.81	31.91	26.6	32.63	26.41	32.88	30.14	39.15	33.36	37.34	27.38	34	27.07	33.29
ISR-21^{-2}	BSR	NA	NA	20.19	78.54	27.71	33.4	26.01	34.57	27.19	31.86	26.04	31.79	27.18	35.17	26.79	32.66	30.78	34.45	33.51	37.31	27.21	34	26.99	34.75
ISR-21^{-3}	BSR	NA	NA	23.59	78.38	31.47	38.07	30.28	36.21	30.94	NA	29.46	NA	30.52	34.38	31.1	34.38	34.53	NA	39.22	NA	30.54	36.79	30.84	NA
ISR-21^{-3}	BSR	NA	NA	23.43	78.38	32.06	NA	30.15	NA	30.99	NA	29.64	35.59	31.3	NA	30.4	33.9	34.53	NA	39.12	NA	30.53	35.5	30.36	NA
ISR-21^{-4}	BSR	NA	NA	28.46	78.78	38.14	NA	37.66	NA	NA	NA	35.54	32.34	NA	41.08	36.02	NA	NA	NA	NA	NA	36.58	38.56	NA	37.52
ISR-21^{-4}	BSR	NA	NA	28.79	77.78	36.83	NA	37.42	NA	NA	NA	36.76	35.01	35.85	NA	36.77	NA	NA	NA	NA	NA	36.57	NA	37.51	NA
ISR-21^{-5}	BSR	NA	NA	31.32	77.47	NA	NA	NA	NA	39.59	NA	NA	NA	NA	NA	42.08	NA	42.53	NA	NA	NA	NA	NA	NA	NA
ISR-21^{-5}	BSR	NA	NA	34.96	77.47	NA	NA	39.82	NA	NA	NA	NA	NA	NA	NA	NA	NA	NA	NA	NA	NA	38.2	NA	NA	NA
ISR-21^{-6}	BSR	NA	NA	31.62	78.38	NA	NA	NA	NA	NA	NA	NA	NA	NA	NA	NA	NA	NA	NA	NA	NA	NA	NA	NA	NA
ISR-21^{-6}	BSR	NA	NA	NA	NA	NA	NA	NA	NA	NA	NA	NA	NA	NA	NA	NA	NA	NA	NA	NA	NA	NA	NA	NA	NA
ISR-21^{-7}	BSR	NA	NA	NA	NA	NA	NA	NA	NA	NA	NA	NA	NA	NA	NA	NA	NA	NA	NA	NA	NA	NA	NA	NA	NA
ISR-21^{-7}	BSR	NA	NA	NA	NA	NA	NA	NA	NA	NA	NA	NA	NA	NA	NA	NA	NA	NA	NA	NA	NA	NA	NA	NA	NA
ISR-23^{-2}	BSR	32.67	30.44	19.75	79.44	25.87	33.53	22.94	NA	24.32	28.56	21.68	27.51	24.99	30.13	22.82	28.58	26.32	NA	27.81	32.89	25.17	31.69	23.78	31.72
ISR-23^{-2}	BSR	32.37	30.72	23.66	79.15	24.95	34.26	21.95	NA	23.82	30.2	21.89	29.58	24.78	27.3	22.88	29.44	27.52	NA	27.34	32.66	24.78	31.6	23.65	31.7
ISR-23^{-3}	BSR	36.43	34.07	22.91	79.44	29.31	36.19	26.8	NA	28.24	36.89	26.44	34.53	28.87	35.94	26.92	32.5	31.95	NA	32.4	37.51	24.87	36.35	27.94	34.74
ISR-23^{-3}	BSR	37.46	33.88	22.99	79.44	29.29	NA	26.13	NA	27.8	33.85	25.37	31.19	29	NA	27.24	37.14	32	NA	31.89	35.98	29.31	34.92	28.44	35.15
ISR-23^{-4}	BSR	NA	NA	26.77	79.44	32.88	NA	30.4	NA	31.4	36.69	29.47	NA	33.09	NA	31.7	NA	36.81	NA	37.01	NA	32.65	37.02	32.18	NA
ISR-23^{-4}	BSR	NA	35.49	26.7	79.43	33.03	NA	30.67	NA	31.37	NA	29.81	NA	32.78	NA	31.75	NA	36.78	NA	37.62	NA	32.96	36.4	31.77	34.78
ISR-23^{-5}	BSR	NA	NA	30.17	79.59	35.71	NA	35.11	NA	34.65	NA	33.58	NA	NA	NA	33.65	NA	39.25	NA	42.42	NA	36.12	NA	35.43	34.59
ISR-23^{-5}	BSR	NA	NA	29.41	79.59	35.89	NA	34.74	NA	34.8	NA	33.29	NA	38.49	NA	34.96	NA	41.14	NA	NA	NA	34.85	NA	NA	31.45
ISR-23^{-6}	BSR	NA	NA	35.3	79.15	36.21	NA	35.6	NA	36.86	NA	36.5	NA	NA	NA	NA	NA	NA	NA	NA	NA	NA	NA	NA	NA
ISR-23^{-6}	BSR	NA	NA	NA	NA	NA	NA	NA	NA	37.5	NA	35.56	NA	NA	NA	36.93	NA	NA	NA	41.66	44.67	37.66	35.47	NA	32.19
ISR-23^{-7}	BSR	NA	NA	NA	NA	NA	NA	NA	NA	NA	NA	NA	NA	NA	NA	NA	NA	NA	NA	NA	NA	NA	NA	NA	40.24
ISR-23^{-7}	BSR	NA	NA	NA	NA	NA	NA	NA	NA	NA	NA	NA	NA	NA	NA	NA	NA	NA	NA	NA	NA	NA	NA	NA	NA

Stram- assay designed by Stram et al. [21]; Erster- assay designed by Erster et al.[17]; Sensi- SensiFAST SYBR® No-ROX Kit (Bioline) was used for Erster et al. assay [17]; all the last- comparative tests of Mix-2 and Mix-8 recently validated assays; Ct- cycle threshold; NA- not amplified; Tm- melting temperature; ß -ACT- ß- actin; Quanta - qScript XLT One-Step RT-qPCR ToughMix(Quantabio); AgPath- AgPath-ID™ One-Step RT-PCR Kit (Life technologies); Clara- Clara™ Probe 1-Step Mix No-ROX (PCR biosystems); Promega- GoTaq 1-Step RT-qPCR System (Promega); Takara- One Step PrimeScript™ RT-PCR Kit (Takara Bio Inc.). AUS-Australian strain BB7721; ISR-21- Israeli ISR-3180/1/21 strain; ISR-23- Israeli ISR-1520/23 strain.

Table S2b. Raw data on evaluation tests of the limit of detection (LOD) based on Log10 dilution series in bovine whole blood of the BEFV strains.

		RT-qPCR kit and system															
		Stram		Erster		Quanta		Quanta		AgPath		AgPath		Clara		Clara	
sample	source	Ct	b-ACT	Ct	Tm	MIX2	b-ACT	MIX8	b-ACT	MIX 2	b-ACT	MIX 8	b-ACT	MIX 2	b-ACT	MIX 8	b-ACT
AUS undel	Vero	18.93	23.85	20.94	79.67	21.3	27.27	18.93	26.67	18.71	26.2	17.28	26.3	18.66	25.36	18.77	25.09
AUS undel	Vero	18.65	22.68	20.85	79.97	20.92	26.78	18.57	26.36	18.72	26.11	17.56	26.56	20.15	25.74	17.51	26.61
AUS 10-1	w.b.	25.1	22.44	26.83	79.67	27.22	22.64	24.89	22.78	23.64	21.09	23.47	21.89	25.35	22.37	24.67	24.12
AUS 10-1	w.b.	25.11	22.53	27.5	79.67	27.21	22.59	24.53	22.42	23.53	21.57	23.08	21.67	25.89	22.84	24.27	23.77
AUS 10-^{2}	w.b.	27.93	22.42	30.29	79.83	30.77	23.03	28.05	23.06	28.0	21.54	26.57	21.64	29.26	23.52	27.96	24.04
AUS 10-^{2}	w.b.	27.98	22.43	30.38	79.68	29.56	21.65	28.31	22.73	28.39	21.75	26.42	21.87	29.59	23.04	28.2	24.37
AUS^{-3}	w.b.	31.24	21.78	36.14	82.94	34.19	22.48	30.78	22.19	30.51	21.46	29.9	21.47	32.69	22.18	30.85	23.35
AUS^{-3}	w.b.	31.13	22.05	33.17	79.51	34.62	22.33	30.15	22.62	30.82	20.53	29.66	21.37	32.54	22.49	31.09	23.75
AUS^{-4}	w.b.	33.56	21.62	40.18	NA	38.56	22.28	36.0	23.0	33.42	21.5	33.34	21.29	36.03	22.18	33.27	23.05
AUS^{-4}	w.b.	36.5	22.11	37.33	82.95	37.05	21.91	35.84	22.61	35.3	21.02	33.28	21.75	35	22.24	33.49	23.68
AUS^{-5}	w.b.	NA	22.43	40.23	NA	NA	22.64	36.79	23.67	NA	21.87	NA	21.82	NA	22.77	36.64	23.36
AUS^{-5}	w.b.	NA	22.52	40.41	NA	NA	22.56	NA	24.16	37.16	21.69	NA	21.59	NA	22.69	NA	23.19
AUS^{-6}	w.b.	NA	22.71	38.51	NA	NA	22.85	NA	23.11	NA	21.63	NA	21.45	NA	22.92	NA	23.49
AUS^{-6}	w.b.	NA	22.78	36.65	NA	NA	22.8	NA	22.0	NA	21.58	NA	22.08	NA	22.82	NA	23.65
ISR-1520/23 undel	Vero	22.5	23.1	22.13	80.12	19.87	23.75	17.38	23.48	17.66	21.55	15.52	23.3	18.87	22.93	16.93	23.01
ISR-1520/23 undel	Vero	22.6	22.96	22.09	80.12	19.39	24.03	17.43	24.62	17.54	21.77	15.44	23.62	18.33	23.39	16.24	23.58
ISR-1520/23 10^{-1}	w.b.	27.1	22.03	26.21	79.82	24.04	21.9	21.99	22.03	21.92	23.72	20.76	21.3	23.56	22.74	21.98	23.7
ISR-1520/23 10^{-1}	w.b.	27.22	22.3	26.08	79.82	26.23	21.31	22.05	24.0	22.0	23.77	20.41	21.45	23.64	22.67	21.98	23.32
ISR-1520/23 10^{-2}	w.b.	30.59	22.41	29.69	79.83	28.01	21.86	27.36	24.83	26.32	21.19	24.14	21.76	27.35	23.0	25.42	23.95
ISR-1520/23 10^{-2}	w.b.	30.59	22.33	30.03	79.83	32.09	22.77	24.4	21.93	26.35	21.4	24.12	21.9	27.67	22.93	25.96	23.82
ISR-1520/23 10^{-3}	w.b.	35.21	22.92	33.73	82.51	32.2	22.77	29.33	22.48	29.58	21.83	27.52	21.97	30.94	23.14	29.22	24.06
ISR-1520/23 10^{-3}	w.b.	34.99	22.86	32.24	82.51	32.09	22.07	31.16	24.37	29.37	21.72	27.37	21.87	31.17	22.84	29.34	23.81
ISR-1520/23 10^{-4}	w.b.	37.45	22.63	39.82	NA	34.91	22.47	33.05	22.12	33.44	22.13	31.09	21.4	33.63	22.52	31.76	22.88
ISR-1520/23 10^{-4}	w.b.	37.76	22.09	32.57	79.97	37.63	23.07	34.03	22.23	33.05	22.28	31.03	21.45	34.26	22.82	32.37	22.6
ISR-1520/23 10^{-5}	w.b.	NA	22.49	37.23	82.52	NA	22.74	36.37	22.66	NA	21.8	35.75	22.28	34.74	22.8	36.09	23.89
ISR-1520/23 10^{-5}	w.b.	NA	22.49	38.88	82.66	38.63	23.44	36.29	23.46	NA	21.46	35.62	21.61	36.67	23.28	37.07	24.05
ISR-1520/23 10^{-6}	w.b.	NA	22.92	36.18	82.53	NA	24.13	NA	23.95	NA	22.12	NA	21.88	NA	23.21	NA	23.92
ISR-1520/23 10^{-6}	w.b.	NA	22.53	37.03	82.53	NA	22.9	NA	22.83	NA	22.35	35.53	21.82	NA	22.9	NA	24.08
w.blood	w.b.	NA	22.22	40.03	NA	NA	22.97	NA	22.97	NA	21.55	NA	21.85	NA	23.02	NA	23.47
w.blood	w.b.	NA	21.84	37.65	82.94	NA	23.06	NA	22.28	NA	21.77	NA	21.78	NA	23.19	NA	23.94

Stram- assay designed by Stram et al. [21]; Erster- assay designed by Erster et al.[17]; Sensi- SensiFAST SYBR® No-ROX Kit (Bioline) was used for Erster et al. assay [17]; all the last- comparative tests of Mix-2 and Mix-8 recently validated assays; Ct- cycle threshold; NA- not amplified; Tm- melting temperature; β -ACT- β- actin; Quanta - qScript XLT One-Step RT-qPCR ToughMix(Quantabio); AgPath- AgPath-ID™ One-Step RT-PCR Kit (Life technologies); Clara- Clara™ Probe 1-Step Mix No-ROX (PCR biosystems). AUS-Australian strain BB7721. Vero-Vero cells; w.blood and w.b.- whole blood.

Table S3. Raw data on diagnostic sensitivity and diagnostic specificity tests positive field samples performed in duplicates.

		RT-qPCR kit and system																							
		Stram		Erster		Quanta		Quanta		AgPath		AgPath		Clara		Clara		TaKaRa		TaKaRa		Promega		Promega	
sample	source	BEFV	b-ACT	BEFV	Tm	MIX2	b-ACT	MIX8	b-ACT	MIX2	b-ACT	MIX8	b-ACT	MIX2	b-ACT	MIX8	b-ACT	MIX2	b-ACT	MIX8	b-ACT	MIX2	b-ACT	MIX8	b-ACT
2119/23	w. b.	34.88	18.57	26.37	80.08	22.22	20.02	20.82	20.05	24.33	18.01	21.38	18.78	26.14	20.62	23.19	21.35	26.99	19.75	25.16	19.71	26.31	22.93	24.29	24
2119/23	w. b.	35.49	18.69	26.37	80.08	22.39	20.11	20.66	20.04	24.94	18.6	21.32	18.84	25.8	20.5	23.29	21.05	26.99	19.57	26.06	20.07	26.51	23.2	24.26	23.79
2120/23	w. b.	NA	18.95	34.72	83.1	NA	20.11	NA	20.2	37.19	19.42	34.92	19.21	NA	20.61	35.24	21.21	39.06	19.26	40.63	20.12	NA	22.86	35.74	24.39
2120/23	w. b.	NA	18.6	36.56	83.39	NA	20.1	36.19	20.19	NA	18.22	34.53	19.22	NA	17.89	36.09	21.64	39.06	19.33	35.01	18.86	NA	23.42	33.14	23.95
2123/23	w. b.	25.42	18.62	NT	NT	24.15	20.67	22.93	20.64	23.17	19.25	20.08	18.94	23.25	20.58	20.93	21.67	24.63	19.18	23.1	19.23	23.74	22.68	22.15	23.92
2123/23	w. b.	25.02	18.36	NT	NT	24.24	20.67	22.86	20.61	22.97	18.64	20.09	19.01	23.38	20.52	20.78	20.91	24.63	18.7	23.53	19.59	23.51	22.45	21.94	23.85
2190/1/23	w. b.	NA	17.4	35.08	82.95	NA	21.87	36.36	21.98	NA	18.13	NA	19.33	37.87	19	36.42	19.97	NA	18.7	NA	18.81	NA	21.76	NA	23.06
2190/1/23	w. b.	NA	17.43	39.5	83.25	NA	21.74	NA	21.81	NA	18.03	35.52	19.33	NA	18.93	36.38	19.92	NA	18.97	43.13	19.21	NA	21.88	39.46	22.81
2190/2/23	w. b.	NA	18.8	34.72	82.94	NA	21.97	NA	21.97	34.17	20	37.13	19.33	37.19	20.57	NA	20.42	NA	19.21	35.82	17.07	NA	23.46	NA	23.16
2190/2/23	w. b.	NA	17.58	37.81	83.39	NA	22.14	NA	23.38	NA	18.12	36.69	23.39	37.6	20.01	NA	19.85	NA	19.21	NA	19.47	NA	23.24	38.49	24.03
2202/23	s+l	24.4	22.54	26.91	80.38	22.59	24.95	21.35	21.97	21.54	24.15	17.89	23.39	21.71	25.52	19.47	26.38	23.31	21.76	21.58	22.2	21.66	27.64	20.52	28.8
2202/23	s+l	24.06	22.53	26.18	80.08	21.72	22.14	21.3	22.38	21.74	23.28	17.89	23.39	20.53	54.56	19.18	26.26	22.97	21.65	20.41	22.6	21.59	26.81	18.89	24.36
2203/23	s+l	NA	17.78	38.98	82.94	NA	23.09	36.5	23.73	NA	23.18	34.03	23.08	34.28	21.23	32.95	21.75	NA	17.9	36.42	17.89	NA	23.66	36.34	24.21
2203/23	s+l	NA	17.43	39.55	83.09	NA	21.71	35.39	21.98	NA	17.57	34.77	23.08	34.86	20.91	32.38	21.18	NA	18.03	37.56	18.11	NA	23.07	37.69	23.81
2205/23	w. b.	39.92	21.89	35.68	83.1	NA	30.38	36.36	23.73	36.85	22	35.64	23.08	36.59	23.71	34.35	24.28	41.31	22.46	38.1	22.62	38.65	26.53	35.87	27.51
2205/23	w. b.	NA	22.16	37.69	83.1	NA	30.95	NA	21.82	NA	26.19	34.03	23.08	37.27	23.74	NA	24.58	NA	22.47	39.51	22.64	NA	26.46	36.17	25.24
2206/1/23	w. b.	36.23	18.65	33.13	82.65	32.14	22.86	36.5	22.86	36.84	20.21	34.74	19.33	35.35	21.41	34	19.6	38.03	20.08	33.83	16.83	37.5	23.83	33.05	21.98
2206/1/23	w. b.	NA	19.75	33.42	84.58	NA	23.09	36.5	22.38	35.93	20.15	33.63	19.33	35.62	21.21	34	19.01	36.84	20.18	35.36	19.85	38.77	23.93	35	23.75
2209/23	s+l	36.79	20.12	NT	NT	29.11	21.88	26.36	20.98	30.54	22.81	28.47	21.33	30.71	22.21	28.12	23.05	32.45	19.09	31.84	19.74	30.54	25.39	28.52	26.14
2209/23	s+l	40.25	20.37	NT	NT	29.69	21.85	26.02	21.24	30.48	22.76	28.47	23.08	25.15	20.29	28.19	23.13	31.69	19.12	29	18.52	29.86	24.8	27.51	23.99
2232/23	w. b.	NA	20.15	35.68	82.94	NA	23.42	38.14	23.73	37.87	20.83	38.17	19.33	37.87	21.67	34.69	22.4	38.8	20.64	39.35	20.5	NA	24.48	35.27	25.8
2232/23	w. b.	NA	20.33	36.69	82.94	NA	25.59	36.5	21.97	31.02	17.64	35.51	19.33	36.13	21.82	35.66	22.42	NA	20.34	39.56	20.73	NA	24.4	NA	25.12
2271/1/23	w. b.	NA	20.2	35.68	82.79	NA	19.76	NA	20.78	NA	20.3	NA	20.44	NA	20.25	NA	19.12	NA	20.91	NA	20.74	NA	22.59	NA	21.74
2271/1/23	w. b.	NA	19.8	35.2	83.09	NA	20.53	NA	21.87	NA	20.67	NA	20.66	NA	20.17	NA	20.96	NA	20.44	NA	20.44	NA	23.67	NA	24.62
2271/2/23	w. b.	23.34	20.52	25.65	79.94	21.96	21.87	20.82	22.97	20.76	20.67	19.64	20.66	23.19	21.02	20.74	21.67	23.5	20.91	22.56	20.28	24.29	24.62	21.93	24.62
2271/2/23	w. b.	23.4	20.58	24.38	29.94	21.89	20.78	20.42	21.87	20.76	20.92	19.64	20.44	22.06	20.48	20.79	21.34	23.59	20.74	21.68	21.75	23.93	23.67	20.83	23.67
2274/1/23	w. b.	21.43	18.64	23.52	79.33	21.96	20.78	18.7	20.78	18.75	18.92	17.78	19.58	20.84	19.75	18.89	20.4	20.9	19.39	20.12	20.69	21.93	22.59	19.82	22.34
2274/1/23	w. b.	21.42	19.17	22.77	79.64	20.82	19.76	18.72	20.78	18.75	19.53	17.78	19.51	21.36	20.09	18.78	19.38	21.66	19.39	20.15	19.15	21.93	22.59	19.82	23.61
2275/1/23	w. b.	22.46	20.46	23.52	80.09	21.89	22.97	20.42	21.87	19.92	20.67	19.49	20.44	23.67	24.33	19.71	21.54	21.82	20.28	21.66	20.91	22.76	23.67	20.83	24.54
2275/1/23	w. b.	22.66	20.29	26.18	80.09	20.82	22.87	20.42	22.89	19.92	20.67	18.67	20.66	22.25	21.64	19.87	21.74	21.97	20.91	21.35	20.91	22.76	23.67	20.83	23.96
80/23	w. b.	NA	17.23	39.06	83.09	NA	20.78	NA	19.76	NA	19.53	NA	19.58	NA	19.66	NA	19.52	NA	19.39	NA	19.39	NA	22.59	NA	21.69
2280/23	w. b.	NA	18.42	34.62	82.94	NA	19.76	NA	20.78	NA	19.07	NA	19.51	NA	19.52	NA	19.92	NA	19.39	NA	NA	NA	22.93	NA	22.51
2286/23	s.	NA	25.89	35.68	82.64	33.89	23.82	NA	27.82	37.8	26.39	36.42	26.39	NA	26.86	NA	27.1	39.73	25.44	38.88	25.44	NA	30.71	NA	30.82
2286/23	s.	NA	25.57	36.56	82.94	NA	27.82	37.29	27.82	36.62	26.39	37.45	28.32	39.42	26.84	NA	26.34	NA	25.44	NA	25.44	NA	31.48	NA	31.63
2250/14	b.c.	NA	27.64	29.94	82.8	30.93	30.36	30.09	29.51	29.3	27.52	28.67	28.24	30.95	28.62	30.15	28.79	31.12	27.34	32.53	27.51	31.89	32.54	30.93	31.63

2250/14	b.c.	NA	27.84	31.76	78.43	29.77	28.42	29.91	29.51	28.79	28.36	28.67	28.24	30.98	28.66	29.99	29.01	31.12	27.34	32.53	27.25	31.89	32.54	30.65	32.46
2267/14	b.c.	NA	21.45	34.63	82.65	30.32	22.71	30.93	23.82	29.97	22.52	30.8	22.44	33.7	22.83	32.99	23.43	37.73	22.14	35.6	21.14	40.48	25.92	32.55	25.74
2267/14	b.c.	NA	22.08	31.91	82.65	30.2	21.87	30.93	23.82	30.29	22.52	30.8	22.44	33.84	22.55	32.82	22.67	NA	21.4	35.15	21.79	38.81	25.8	32.55	25.86
1747/18	b.c.	NA	20.39	NT	NT	31.81	22.89	30.09	23.82	30.29	20.42	29.41	20.44	35.16	23.7	31.4	21.77	33.17	21.4	37.73	21.36	34.06	23.67	31.89	24.54
1747/18	b.c.	NA	20.43	NT	NT	31.81	22.89	30.32	23.82	30.29	20.58	29.42	20.44	34.14	22.59	31.67	21.92	32.53	23.62	36.85	21.8	34.91	23.67	31.89	24.8
1847/5/18	b.c.	NA	26.77	34.79	78.73	30.17	24.79	30.32	26.81	29.79	22.52	28.45	22.44	33.69	24.25	30.6	24.58	32.22	23.62	36.36	23.7	32.55	25.92	30.65	26.95
1847/5/18	b.c.	NA	26.8	31.76	78.58	30.17	24.79	30.05	28.42	28.67	21.87	28.67	22.2	34.05	24.82	30.43	24.79	32.22	24.09	35.15	23.62	33.09	25.92	30.65	26.95
1958/18	b.c.	NA	19.32	20.55	78.43	21.96	20.1	18.43	19.85	17.94	17.42	17.77	18.17	21.79	21.69	18.71	20.36	21.66	19.39	21.66	20.06	21.93	21.63	20.83	22.89
1958/18	b.c.	NA	20.06	20.77	78.43	21.96	20.1	18.05	19.72	17.94	17.42	17.24	18.17	21.6	19.44	NT	NT	21.68	19.39	21.68	19.27	21.93	21.76	20.83	22.89
2218/4/23	w. b.	21.39	21.56	17.29	79.81	19.99	19.33	18.48	20.88	19.85	20.39	18.92	20.92	17.75	18.34	17.69	19.86	21.55	18.16	20.08	19.39	19.3	22.27	17.51	22.47
2218/4/23	w. b.	22.44	21.49	16.95	79.95	19.88	18.84	18.2	20.8	19.79	20.46	18.83	20.64	16.77	18.32	17.3	19.06	21.73	18.99	20.02	19.43	19.26	22.18	17.53	22.94
2206/2/23	w. b.	29.98	22.58	24.68	79.51	28.04	20.84	26.61	22.39	27.44	22.44	27.02	22.72	23.14	19.26	22.81	20.55	31.49	20.73	32.08	20.91	27.57	24.67	26.15	25.26
2206/2/23	w. b.	30.26	22.59	25.28	79.51	27.65	20.79	26.34	22.35	27.28	21.91	26.88	22.37	19.88	19.35	23.33	20.42	28.53	18.75	32.15	21.34	26.41	23.64	25.75	24.93
2210/23	w. b.	30.81	22.59	24.43	79.95	28.53	21.41	26.61	22.61	27.67	22.27	26.75	22.37	23.99	16.26	21.16	19.89	30.7	19.49	30.13	20.48	27.91	24.55	25.87	25.06
2210/23	w. b.	30.44	22.6	24.73	79.95	28.23	20.79	26.5	22.27	26.96	21.58	26.82	22.46	23.6	19.88	22.44	21.13	31.33	20.71	30.69	20.22	27.73	24.71	25.84	25.11
2213/23	w. b.	25.66	23.15	19.3	79.81	23.66	21.97	21.48	23.64	23.03	22.77	22.07	23.35	20.13	21.04	20.09	20.37	25.41	21.3	23.77	21.46	22.53	24.73	20.99	26
2213/23	w. b.	26.1	23.28	19.09	80.11	23.67	21.94	21.75	23.7	23.09	22.69	22.06	23.13	20.83	21.15	21.01	21.42	25.66	21.81	24.16	21.58	22.49	25.14	20.64	25.8
3313/1/21	b.c.	NA	28.15	14.44	77.99	21.34	26.17	19.96	29.28	20.49	27.14	19.57	27.45	17.14	24.47	17.57	26	21.93	24.58	25.7	26.8	19.6	27.77	17.67	28.94
3313/1/21	b.c.	NA	28.78	14.34	77.68	21.27	27.03	19.61	29.4	20.34	27.16	19.5	27.18	16.34	23.97	17.64	27.14	22.25	25.74	25.42	26.75	19.23	28.99	18.42	29.23
3330/21	b.c.	NA	27.73	15.55	77.99	21.3	26.39	19.37	28.56	20.7	27.32	19.42	27.38	17.82	25.16	17.92	26.06	22.82	25.6	25.19	26.52	19.71	29.93	18.53	29.55
3330/21	b.c.	NA	26.61	16.74	77.99	21.3	26.8	19.17	28.46	20.43	26.57	18.88	25.77	18.06	24.98	17.03	25.56	22.76	25.89	21	19.81	19.6	29.45	17.78	29.9
2285/2/21	b.c.	NA	28.73	22.88	77.99	22.88	27.96	20.9	29.49	21.89	27.22	20.86	27.15	16.6	25.61	20.29	26.57	23.43	24.54	25.41	25.99	21.02	30.01	19.96	30.19
2285/2/21	b.c.	NA	28.25	22.9	77.83	22.57	26.67	20.84	29.84	21.93	27.11	20.95	27.35	20.35	25.88	19.51	26.25	23.89	25.25	25.93	26.35	20.67	29.42	19.62	30.1
3368/1/21	b.c.	NA	25.93	22.83	77.98	27.22	25.26	25.81	26.94	26.43	26.17	25.34	26.26	25.9	24.94	24.72	23.81	29.25	24.75	33.21	25.39	26.15	28.41	24.72	28.89
3368/1/21	b.c.	NA	25.98	23.14	77.98	22.38	25.31	25.66	27.44	26.45	26.42	25.55	26.31	26.05	25.61	24.66	23.69	29.9	25.15	33.85	25.61	25.79	28.36	24.12	28.17
3386/21	p.	NA	25.79	25.44	77.99	32.37	24.95	30.61	24.64	31.68	26.4	28.78	24.44	30.69	23.7	27.51	22.99	35.22	23.94	29.97	18.44	31.37	29	28.26	28.67
3386/21	p.	NA	26.96	25.52	78.14	32	25.32	29.7	26.64	29.88	25.19	28.67	24.49	31.16	23.93	22.86	22.41	35.54	22.82	37.17	24.47	31.18	29.36	29.83	29.43
3386/21	b.c.	NA	27.1	22.83	77.99	29.69	26.71	27.63	28.26	29.05	27.95	27.41	27.6	24.31	24.73	25.35	25.48	32.21	26.45	35.46	26.7	28.4	30.19	27.46	30.85
3386/21	b.c.	NA	27.63	23.14	77.99	29.49	26.83	28.02	27.8	28.97	28.98	25.22	24.76	25.85	25.79	24.85	24.73	32.04	26.28	32.97	26.18	27.84	30.15	26.91	30.59
3038/3/21	b.c.	NA	30.08	16.4	78.14	23.84	28.57	22.1	30.49	23.25	28.5	21.22	28.09	20	26.91	20.17	26.81	25.3	26.24	26.93	27.19	22.21	30.75	21.42	31.19
3038/3/21	b.c.	NA	29.2	16.64	78.14	23.62	28.12	22.06	30.61	23.08	28.56	22.07	28.61	19.9	26.22	19.64	26.98	25.14	26.58	27.19	27.58	22.12	30.64	21.37	31.46
1900/18	b.c.	NA	27.97	22.55	78.29	28.82	25.85	27.37	27.68	28.18	27.13	27.42	27.25	22.32	25.2	23.43	25.62	31.08	24.65	34.25	25.62	27.66	29.67	25.91	30.01
1900/18	b.c.	NA	28.6	22.61	78.29	28.54	25.72	27.45	27.53	28.39	27.41	27.38	27.12	25.54	25.31	25.34	25.01	31.12	24.68	33.43	25.01	28.14	29.96	26.93	30.37
1934/1/18	b.c.	NA	25.25	15.24	78.14	21.97	22.99	19.56	23.74	21.34	24.03	16.76	19.25	18.91	22.29	17.76	22.59	21.8	19.74	23.61	21.42	20.42	25.81	19.3	23.98
1934/1/18	b.c.	NA	25.35	15.55	77.99	21.49	22.02	19.84	23.77	21.37	24.09	18.93	22.15	18.31	22.47	18.51	22.45	21.7	21.75	24.62	22.39	20.13	25.67	18.83	24.64
1813/11	b.c.	NA	26.61	33.34	82.92	30.94	27.42	28.08	26.91	31.94	26.42	31.27	25.99	33.06	28.34	32.39	26.73	32.83	27.49	38.13	28.43	30.15	27.83	29.09	27.79
1813/11	b.c.	NA	26.71	34.09	83	31.64	27.61	27.86	26.74	31.78	26.51	31.44	26.52	33.35	28.38	32.98	27.23	33.26	27.14	39.02	28.48	31.54	28.01	28.86	27.91

2068/1/14	b.c.	NA	25.74	33.85	82.93	31.78	26.37	30.69	26.89	31.71	25.84	31.49	26.03	32.29	26.92	32.84	25.43	30.63	25.41	39.35	26.38	31.09	28.1	30.89	26.36
2068/1/14	b.c.	NA	26.63	34.15	82.64	31.78	26.63	29.57	26.84	31.88	25.61	31.09	24.8	32.9	26.97	32.61	25.13	28.58	24.57	38.32	25.93	30.96	27.8	30.44	26.6
2068/2/14	b.c.	NA	28.93	37.37	82.42	30.53	31.33	27.16	30.24	29.57	28.22	28.8	27.97	30.96	29.82	28.34	28.03	30.9	27.14	32.75	29.46	28.19	28.86	27.41	29.56
2068/2/14	b.c.	NA	29.04	35	82.48	30.03	29.89	26.9	29.6	27.81	27.14	28.17	28.09	30.59	29.76	28.44	27.73	30.69	27.96	31.9	29.27	29.03	29.08	27.66	28.6
2068/3/14	b.c.	NA	23.78	NA	82.9	27.94	24.96	25.89	24.04	27.14	22.87	27.76	22.89	28.03	24.93	28.73	23.73	27.8	22.38	33.52	23.65	26.52	24.13	24.98	23.37
2068/3/14	b.c.	NA	23.94	36.47	78.01	27.22	24.06	27.09	25.77	27.59	23.95	28.19	23.82	27.74	24.3	28.84	24.14	28.46	22.61	32.78	23.55	24.05	25.17	24.92	22.81
2078/1/14	b.c.	NA	22.02	24.9	78.03	18.85	21.01	22.34	22.46	20.97	20.71	23.11	20.25	21.81	22.91	23.65	21.09	19.63	20.92	27.55	21.94	20.56	23.4	22.36	20.86
2078/1/14	b.c.	NA	22.04	25.8	78.03	20.4	21.46	21.43	22.19	21	20.27	24.43	20.55	22.01	22.74	23.5	20.95	21.03	20.89	27.85	21.89	20.71	23.29	22.39	20.9
2078/3/14	b.c.	NA	25.41	30.86	78.18	27.88	27.05	22.37	25.73	26.68	24.31	24.43	23.3	27.2	25.87	25.91	23.96	26.87	24.21	30.02	25.14	20.64	26.29	23.56	23.9
2078/3/14	b.c.	NA	25.47	30.22	78.18	25.96	26.65	24.47	26.16	26.73	24.01	25.07	23.44	27.24	25.67	25.75	24.6	27.13	24.19	30.08	25.24	25.53	26.76	NA	NA
1631/11	b.c.	NA	28.47	32.87	82.92	31.72	29.4	27.9	28.78	31.56	27.47	30.19	27.21	32.67	28.47	31.31	26.73	34.98	30.46	35.44	30.44	31.21	29.64	31.05	30.15
1631/11	b.c.	NA	28.2	31.81	83.21	31.69	29.67	27.13	28.52	31.39	27.75	30.31	27.71	32.54	28.5	30.81	26.72	35.33	30.78	35.33	31.45	31.31	29.28	30.5	27.94
2073/13	b.c.	NA	27.81	34.39	82.48	26.67	30.85	26.98	30.36	28.89	29.26	26.76	28.71	27.49	28.41	28.84	28.34	31.28	30.13	32.47	30.39	26.73	31.1	25.27	29.28
2073/13	b.c.	NA	28.78	32.42	82.92	26.61	31.01	26	29.71	28.5	28.9	27.61	29.86	27.29	29.54	28.64	28.09	31.46	30.36	32.09	30.43	26.25	30.16	25.08	27.98

Stram- assay designed by Stram et al. [21]; Erster- assay designed by Erster et al.[17]; Sensi- SensiFAST SYBR® No-ROX Kit (Bioline) was used for Erster et al. assay [17]; all the last- comparative tests of Mix-2 and Mix-8 recently validated assays; Ct- cycle threshold; w.b.- whole blood; b.c.- buffy coat; P.- plasma; s.- spleen; s+l- mix of spleen and lung; NA- not amplified; b -ACT- β- actin; Quanta - qScript XLT One-Step RT-qPCR ToughMix(Quantabio); AgPath- AgPath-ID™ One-Step RT-PCR Kit (Life technologies); Clara- Clara™ Probe 1-Step Mix No-ROX (PCR biosystems); Promega- GoTaq 1-Step RT-qPCR System (Promega); Takara- One Step PrimeScript™ RT-PCR Kit (Takara Bio Inc.); Tm- melting temperature.

Table S4. Raw data on diagnostic sensitivity and diagnostic specificity test performed with field samples in one plicate.

		RT-qPCR kit and system															
		Stram		Erster		Quanta		Quanta		AgPath		AgPath		Clara		Clara	
sample	source	Ct	b-ACT	Ct	Melt T	MIX2	b-ACT	MIX8	b-ACT	MIX 2	b-ACT	MIX 8	b-ACT	MIX 2	b-ACT	MIX 8	b-ACT
1293/1/24	w.b.	NA	22.37	36.01	82.0	NA	21.4	NA	23.23	NA	22.94	NA	23.22	NA	23.39	NA	23.85
1293/2/24	w.b.	NA	23.31	35.31	82.0	NA	21.13	NA	22.64	NA	22.86	NA	23.44	NA	23.76	NA	23.88
1293/3/24	w.b.	NA	23.47	37.96	82.0	NA	22.8	NA	21.69	NA	23.52	NA	24.72	NA	24.38	NA	24.46
1294/24	w.b.	NA	22.21	NA	NA	NA	21.97	NA	22.68	NA	22.99	NA	23.36	NA	23.9	NA	24.42
1295/1/24	w.b.	NA	21.51	35.16	82.0	NA	20.43	NA	21.31	NA	21.78	NA	21.8	NA	22.69	NA	22.85
1295/2/24	w.b.	NA	21.53	35.02	82.0	NA	20.49	NA	20.93	NA	22.04	NA	22.2	NA	22.65	NA	23.15
1295/3/24	w.b.	NA	21.03	NA	NA	NA	19.86	NA	21.33	NA	22.06	NA	22.03	NA	22.85	NA	22.34
1295/4/24	w.b.	NA	22.2	40.62	NA	NA	22.87	NA	23.85	NA	23.86	NA	24.17	NA	23.67	NA	24.8
1295/5/24	w.b.	NA	21.57	33.89	82.0	NA	20.54	NA	22.08	NA	23.35	NA	22.85	NA	23.44	NA	23.68
1295/6/24	w.b.	NA	20.81	NA	NA	NA	20.81	NA	21.21	NA	21.9	NA	22.28	NA	22.41	NA	23.01
1295/7/24	w.b.	NA	23.59	39.23	NA	NA	22.17	NA	22.12	NA	23.52	NA	23.6	NA	24.98	NA	24.15
1295/8/24	w.b.	NA	20.9	NA	NA	NA	20.31	NA	20.56	NA	21.3	NA	21.53	NA	22.29	NA	22.42
1295/9/24	w.b.	NA	22.3	36.7	NA	NA	21.83	NA	22.14	NA	22.08	NA	22.23	NA	23.33	NA	23.15
1295/10/24	w.b.	NA	20.62	37.75	82.53	NA	20.03	NA	20	NA	20.79	NA	21.69	NA	21.46	NA	21.88
1295/11/24	w.b.	NA	21.51	40.54	NA	NA	20.54	NA	20.43	NA	21.54	NA	20.97	NA	21.79	NA	21.51
1295/12/24	w.b.	NA	19.18	36.62	82.22	NA	19.58	NA	20.2	NA	20.26	NA	20.24	NA	22.2	NA	20.94
1299/1/24	w.b.	NA	18.42	38.53	NA	NA	17.16	NA	17.44	NA	15.54	NA	15.42	NA	19.02	NA	20.18
1299/2/24	w.b.	NA	17.74	36.75	NA	NA	17.18	NA	16.54	NA	15.39	NA	16.38	NA	17.87	NA	19.46
1299/3/24	w.b.	NA	19.79	34.67	82.0	NA	17.19	NA	17.74	NA	16.61	NA	17.49	NA	17.71	NA	19.64
1299/5/24	w.b.	NA	17.84	41.14	NA	NA	16.99	NA	17.59	NA	16.39	NA	17.18	NA	18.68	NA	19.39
1311/1/24	w.b.	NA	20.87	39.04	82.41	NA	17.12	NA	17.48	NA	18.98	NA	19.94	NA	21.41	NA	22.72
1311/2/24	w.b.	NA	20.3	36.71	82.56	NA	20.44	NA	20.97	NA	18.06	NA	19.14	NA	19.8	NA	21.12
1312/24	w.b.	NA	19.53	39.06	NA	NA	19.71	NA	20.72	NA	17.13	NA	17.9	NA	18.82	NA	19.84
1318/24	w.b.	NA	19.79	NA	NA	NA	18.45	NA	18.96	NA	15.45	NA	19.94	NA	19.29	NA	20.1
1320/1/24	w.b.	NA	21.79	38.74	NA	NA	19.93	NA	20.85	NA	19.01	NA	20.34	NA	20.78	NA	21.65
1320/2/24	w.b.	NA	19.2	NA	NA	NA	18.74	NA	19.63	NA	17.13	NA	18.42	NA	19.32	NA	20.16
1320/3/24	w.b.	NA	20.77	NA	NA	NA	19.14	NA	19.75	NA	17.42	NA	18.66	NA	19.85	NA	20.23
1320/4/24	w.b.	NA	20.33	NA	NA	NA	18.68	NA	19.08	NA	18.22	NA	18.42	NA	20.37	NA	21.19
1322/24	s.	NA	25.95	NA	NA	NA	24.84	NA	25.2	NA	23.22	NA	24.05	NA	23.69	NA	24.88
1326/24	w.b.	NA	20.87	NA	NA	NA	20.98	NA	21.15	NA	18.71	NA	21.29	NA	19.9	NA	20.67
1327/24	w.b.	NA	18.97	NA	NA	NA	18.46	NA	18.64	NA	16.45	NA	16.9	NA	17.25	NA	17.63
1330/1/24	w.b.	NA	21.93	NA	NA	NA	21.17	NA	20.49	NA	18.7	NA	19.46	NA	19.89	NA	20.73
1330/2/24	w.b.	NA	18.45	NA	NA	NA	18.01	NA	17.53	NA	15.68	NA	16.56	NA	16.82	NA	17.96

1324/24	s.	NA	20.95	38.18	NA	NA	23.32	NA	23.37	NA	21.18	NA	22.28	NA	21.18	NA	21.76
1315/24n	w.b.	NA	22.97	40.65	NA	NA	22.89	NA	22.21	NA	19.9	NA	20.65	NA	21.02	NA	21.33
1338/24	w.b.	NA	20.54	NA	NA	NA	21.41	NA	21.33	NA	22.35	NA	23.21	NA	19.48	NA	19.64
1339/24	w.b.	NA	20.87	NA	NA	NA	22.06	NA	22.07	NA	23.64	NA	24.93	NA	20.62	NA	21.13
1340/23	s.	NA	30.67	NA	NA	NA	30.23	NA	30.25	NA	28.18	NA	28.93	NA	28.96	NA	29.82
1341/24	s+l	NA	22.96	NA	NA	NA	23.57	NA	24.04	NA	21.23	NA	21.73	NA	21.72	NA	22.29
1347/1/24	w.b.	NA	21.21	NA	NA	NA	22.08	NA	21.96	NA	18.63	NA	20.49	NA	22.18	NA	23.03
1347/2/24	w.b.	NA	22.11	40.23	NA	NA	23.12	NA	23.07	NA	20.29	NA	21.12	NA	23.15	NA	23.67
1347/3/24	w.b.	NA	21.58	38.37	82.0	NA	22.18	NA	22.93	NA	19.7	NA	20.68	NA	22.11	NA	23.01
1347/4/24	w.b.	NA	20.2	NA	NA	NA	20.35	NA	20.42	NA	16.65	NA	19.84	NA	21.26	NA	21.69
1347/5/24	w.b.	NA	22.22	NA	NA	NA	23.07	NA	20.31	NA	18.84	NA	21.38	NA	22.48	NA	23.38
1347/6/24	w.b.	NA	22.13	NA	NA	NA	23.33	NA	21.7	NA	20.5	NA	21.04	NA	22.54	NA	23.28
1347/7/24	w.b.	NA	22.67	NA	NA	NA	23.59	NA	23.15	NA	20.11	NA	21.64	NA	22.58	NA	23.43
1347/8/24	w.b.	NA	21.91	36.54	82.5	NA	22.75	NA	21.9	NA	19.21	NA	20.6	NA	22.33	NA	22.53
1347/9/24	w.b.	NA	18.38	35.33	75.29	NA	21.43	NA	20.24	NA	18.44	NA	19.5	NA	20.99	NA	21.45
1347/10/24	w.b.	NA	20.23	NA	NA	NA	21.66	NA	20.95	NA	18.7	NA	19.9	NA	21.48	NA	21.86
1347/11/24	w.b.	NA	19.99	NA	NA	NA	22.13	NA	21.56	NA	19.53	NA	20.13	NA	22.11	NA	22.42
1354/24	w.b.	NA	22.64	NA	NA	NA	22.35	NA	22.06	NA	20.54	NA	21.82	NA	23.14	NA	23.33
1355/1/24	w.b.	NA	23.47	NA	NA	NA	22.8	NA	23.13	NA	22.51	NA	22.58	NA	22.08	NA	23.33
1355/2/24	w.b.	NA	22.86	NA	NA	NA	20.89	NA	22.39	NA	20.95	NA	21.92	NA	22.16	NA	22.46
1355/3/24	w.b.	NA	21.57	40.1	84.3	NA	20.67	NA	21.66	NA	20.19	NA	20.68	NA	20.56	NA	21.07
1356/1/24	w.b.	NA	21.73	37.5	82.35	NA	20.27	NA	21.08	NA	19.84	NA	21.14	NA	20.88	NA	21.11
1356/2/24	w.b.	NA	21.94	37.52	82.35	NA	21.03	NA	21.23	NA	20.31	NA	20.94	NA	21.09	NA	21.23
1356/3/24	w.b.	NA	23.38	39.83	84.31	NA	21.9	NA	22.95	NA	20.97	NA	22.07	NA	22.04	NA	22.61
1356/4/24	w.b.	NA	22.46	NA	NA	NA	20.36	NA	21.75	NA	20.91	NA	21.41	NA	20.89	NA	21.93
1356/5/24	w.b.	NA	23.18	NA	NA	NA	22.6	NA	22.69	NA	21.2	NA	21.01	NA	21.78	NA	22.17
1365/24	w.b.	NA	19.95	34.63	NA	NA	17.33	NA	17.04	NA	21.79	NA	21.47	NA	17.91	NA	18.89
1366/1/24	w.b.	NA	19.84	36.15	80.77	NA	18.11	NA	18.54	NA	17.71	NA	17.7	NA	19.4	NA	20.17
1366/2/24	w.b.	NA	19.09	34.82	83.46	NA	18.33	NA	18.02	NA	17.14	NA	16.68	NA	18.55	NA	18.74
1367/24	s.	NA	21.87	NA	NA	NA	23.41	NA	23.25	NA	19.46	NA	20.49	NA	21.3	NA	21.24
1378/24	w.b.	NA	21.38	37.18	82.62	NA	20.72	NA	21.45	NA	18.12	NA	19.24	NA	18.94	NA	19.77
147/24	s+l	NA	29.73	39.22	74.06	NA	29.21	NA	30.55	NA	27.1	NA	28.12	NA	25.71	NA	25.07
147/24	brain	NA	18.95	34.76	82.0	NA	30.43	NA	30.37	NA	26.46	NA	26.61	NA	28.59	NA	29.5
1352/23	serum	NA	26.59	NA	NA	NA	27.08	NA	27.68	NA	25.36	NA	25.52	NA	26.71	NA	25.93
1373/23	serum	NA	18.9	35.32	83.8	NA	18.46	NA	20.08	NA	17.29	NA	17.1	NA	18.59	NA	18.43
1369/23	s.	NA	26.03	NA	NA	NA	25.3	NA	25.6	NA	21.99	NA	22.87	NA	24.26	NA	24.82
1373/23	serum	NA	28.23	NA	NA	NA	26.71	NA	25.98	NA	22.67	NA	23.7	NA	25.9	NA	25.63

1420/23	brain	NA	28.23	NA	NA	NA	29.6	NA	29.85	NA	28.54	NA	28.83	NA	28.94	NA	29.7
1474/23	s.	NA	28.48	41.66	NA	NA	30.49	NA	30.57	NA	27.72	NA	28.29	NA	21.72	NA	27.37
1478/2/23	w.b.	NA	22.93	NA	NA	NA	23.21	NA	23.3	NA	21.16	NA	21.99	NA	19.74	NA	21.45
1478/3/23	w.b.	NA	22.63	39.6	NA	NA	22.62	NA	21.7	NA	20.67	NA	21.24	NA	19.78	NA	20.75
1620/23	w.b.	NA	20.52	38.88	NA	NA	22.17	NA	23.01	NA	19.72	NA	20.08	NA	20.18	NA	20.64
1620/23	serum	NA	35.01	39.63	NA	NA	37.49	NA	36.98	NA	NA	NA	34.59	NA	32.45	NA	35.29
1480/2/23	w.b.	NA	19.31	36.75	NA	NA	18.61	NA	19.43	NA	17.22	NA	17.71	NA	19.73	NA	20.08
1482/1/23	w.b.	NA	20.06	41.95	NA	NA	22.81	NA	23.12	NA	20.9	NA	21.17	NA	21.48	NA	22.03
**1482/2/23**	**w.b.**	**NA**	**22.48**	**35.75**	**82.0**	**36.18**	**20.67**	**33.44**	**19.1**	**33.91**	**17.68**	**35.67**	**19.57**	**38.01**	**17.97**	**36.71**	**17.94**
1482/4/23	w.b.	NA	24.9	35.22	82.35	NA	23.2	NA	23.13	NA	22.99	NA	23.32	NA	25.36	NA	25.67
1336/23	w.b.	NA	20.81	35.75	NA	NA	19.11	NA	20.65	NA	16.77	NA	17.72	NA	19.88	NA	20.32
1339/1/23	w.b.	NA	19.9	39.11	82.76	NA	19.85	NA	21.21	NA	18.61	NA	19.28	NA	19.58	NA	20.85
1350/1/23	w.b.	NA	21.62	35.59	82.31	NA	19.99	NA	19.61	NA	19.68	NA	19.59	NA	20.88	NA	22.35
**2241/1/23**	**w.b.**	**35.22**	**20.28**	**41.13**	**NA**	**32.89**	**21.43**	**30.47**	**21.1**	**30.82**	**20.55**	**30.33**	**22.19**	**33.45**	**20.91**	**30.61**	**20.45**
**2241/2/23**	**w.b.**	**35.95**	**22.06**	**38.48**	**82.44**	**33.03**	**23.15**	**31.35**	**23.28**	**31.75**	**21.99**	**30.41**	**23.04**	**32.4**	**21.46**	**32.72**	**23.44**
**2045/23**	**brain**	**NA**	**26.94**	**40.77**	**NA**	**37.68**	**29.22**	**34.9**	**29.06**	**34.52**	**26.22**	**33.55**	**27.62**	**36.64**	**29.26**	**36.86**	**29.8**
**3239/09**	**b.c.**	**NA**	**27.28**	**NA**	**81.78**	**31.17**	**26.43**	**29.61**	**26.79**	**30.34**	**28.07**	**28.14**	**28.51**	**33.06**	**29.1**	**29.47**	**28.8**
3183/09	b.c.	NA	28.64	41.52	NA	NA	27.33	NA	28.2	NA	25.94	NA	26.35	NA	28.87	NA	29.51
3170/09	b.c.	NA	24.8	36.92	82.0	NA	23.24	NA	23.76	NA	22.15	NA	22.49	NA	25.11	NA	26.89
3135/2/09	b.c.	NA	31.36	40.76	82.71	NA	30.9	NA	31.55	NA	31.41	NA	30.92	NA	31.23	NA	30.86
3136/09	b.c.	NA	29.3	NA	NA	NA	26.92	NA	29.09	NA	29.38	NA	27.4	NA	27.88	NA	28.43
3378/09	b.c.	NA	31.5	36.65	82.74	NA	31.76	NA	32.14	NA	29.73	NA	29.81	NA	28.59	NA	29.5
3100/1/09	b.c.	NA	31.54	36.88	83.05	NA	33.24	NA	33.77	NA	29.19	NA	29.99	NA	29.62	NA	31.08
3153/09	b.c.	NA	26.5	38.8	82.41	NA	25.62	NA	25.38	NA	25.37	NA	25.7	NA	23.08	NA	20.65
3165/09	b.c.	NA	30.31	38.7	82.87	NA	28.04	NA	28.91	NA	28.22	NA	28.85	NA	25.25	NA	26.2
1608/5/15	b.c.	NA	30.57	36.62	82.71	NA	29.71	NA	30.26	NA	27.9	NA	27.79	NA	29.67	NA	30.91
**2070/14**	**b.c.**	**NA**	**31.55**	**NA**	**NA**	**30.26**	**28.54**	**28.69**	**29.94**	**30.23**	**28.68**	**28.95**	**29.16**	**31.95**	**29.99**	**29.0**	**28.6**
2076/14	b.c.	NA	26.25	37.52	82.4	NA	22.83	NA	23.46	NA	23.04	NA	23.05	NA	23.75	NA	23.51
**2188/1/18**	**b.c.**	NA	31.01	34.64	82.55	NA	32.84	NA	30.28	**38.69**	32.21	NA	32.45	NA	30.39	**35.37**	29.28
3268/09	b.c.	NA	24.3	27.85	78.96	27.15	23.95	26.4	24.31	26.56	24.21	24.26	23.83	27.72	24.89	26.21	24.86
3182/09	b.c.	NA	31.94	28.68	77.98	30.31	31.91	27.33	31.55	25.86	31.64	24.26	32.67	27.6	30.11	26.05	31.78
3184/09	b.c.	NA	26.19	25.91	77.83	28.54	25.53	26.45	25.88	25.98	23.81	22.67	24.23	31.7	27.12	27.06	27.76
2973/09	b.c.	NA	28.6	25.68	77.85	28.17	28.54	25.41	28.68	26.37	27.75	23.79	28.46	28.34	29.68	26.1	29.4
2969/09	b.c.	NA	29.25	27.21	77.85	29.57	28.66	27.28	28.52	27.32	27.75	27.2	29.11	30.02	29.7	25.53	30.48
3030/2/09	b.c.	NA	24.58	22.91	77.7	25.11	24.25	23.19	24.39	23.53	23.01	21.4	24.19	26.02	25.12	22	25.82
3030/3/09	b.c.	NA	25.45	22.91	77.7	25.54	25.06	22.79	25.19	23.64	24.45	22.6	25.0	25.69	26.01	22.42	27
3081/09	b.c.	NA	31.14	27.58	77.67	30.07	31.77	31.39	33.3	27.67	29.82	27.48	31.49	29.61	31.83	27.44	32.61

3094/09	b.c.	NA	26.53	26.87	77.67	29.29	26.25	27.52	26.19	27.92	24.97	25.68	25.71	29.17	27.22	27.72	28.13
3180/09	b.c.	NA	25.37	22.07	77.7	25.64	24.76	23.86	24.99	23.61	22.02	21.43	22.88	24.91	25.76	22.47	26.63
3171/09	b.c.	NA	28.34	28.59	77.7	30.83	28.35	28.76	29.55	28.67	26.35	24.21	26.95	25.87	28.63	26.54	29.84
3173/1/09	b.c.	NA	36.18	28.56	77.67	28.96	24.84	27.5	26.46	27.95	23.94	25.82	24.05	28.93	26.33	26.18	27.2
3173/2/09	b.c.	NA	27.25	28.95	77.82	30.19	26.62	28.17	27.45	28.13	24.72	26.87	25.61	26.33	26.77	26.98	28.52
3340/09	b.c.	NA	28.38	26.52	77.91	26.36	27.5	28.95	27.39	27.19	25.28	25.57	26.26	29.84	28.57	24.37	28.8
1315/24	w.b.	NA	21.61	32.89	82.42	31.61	18.13	34.84	21.54	34.6	19.43	33.7	20.69	35.9	21.23	33.52	22.28
3164/09	b.c.	NA	24.69	28.28	77.96	29.41	23.94	28.01	24.59	29.38	23.5	27.64	23.77	30.39	23.98	28.56	23.56
3170/09	b.c.	NA	14.93	23.35	77.65	25.95	25.62	25.0	25.91	26.13	25.28	24.96	25.7	26.17	23.17	25.28	24.3
3138/09	b.c.	NA	25.9	21.53	77.97	26.97	26.44	23.89	26.86	24.95	23.49	22.44	23.48	27.4	25.96	24.81	26.93
3393/09	b.c.	NA	29.29	29.71	77.97	35.0	29.49	32.04	30.14	31.6	25.29	30.59	26.13	35.91	28.4	33.43	29.08
1957/18	b.c.	NA	21.66	25.89	78.15	31.08	22.51	27.59	22.73	29.11	20.39	27.6	19.95	29.15	21.72	31.83	21.89
1966/1/18	b.c.	NA	29.75	21.81	77.99	26.39	30.05	24.72	30.42	24.58	27.26	24.16	28.21	24.96	29.95	26.58	28.8
2126/18	b.c.	NA	26.16	21.42	77.99	25.26	26.61	25.15	27.67	23.56	24.15	22.22	24.35	24.85	26.25	25.37	25.57
2240/18	b.c.	NA	28.48	29.48	78.14/82.0	32.46	29.91	31.2	30.11	29.16	25.94	29.25	26.92	30.81	28.52	32.9	28.75
2231/18	b.c.	NA	29.26	25.32	78.14	28.78	30.81	26.5	31.53	24.46	24.86	24.0	26.8	28.75	29.2	26.7	29.73
2234/1/18	b.c.	NA	29.66	31.67	78.14/82.0	35.04	30.39	32.31	30.54	31.23	25.81	30.36	26.8	34.84	28.58	32.18	29.03
2234/2/18	b.c.	NA	23.11	21.63	77.99	25.49	23.83	23.16	23.9	22.04	19.21	20.79	19.88	25.95	23.58	23.41	23.46
2112/18	b.c.	NA	26.07	26.86	77.96	31.42	26.14	31.11	27.47	29.15	23.62	28.96	23.65	32.19	27.47	31.77	29.13
2056/2/18	b.c.	NA	31.04	29.82	77.96	34.77	32.52	32.22	33.33	31.5	28.56	29.58	29.34	34.63	32.58	31.71	31.93
2081/18	b.c.	NA	23.93	25.72	77.97	30.33	24.16	30.15	24.48	27.84	20.82	28.0	20.71	30.32	24.77	30.64	26.63
2089/18	b.c.	NA	26.83	22.66	77.97	27.72	26.37	25.82	26.21	26.52	25.47	24.03	25.02	28.26	27.45	25.02	27.63
2127/1/18	b.c.	NA	20.01	17.33	78.12	14.26	14.05	19.62	18.54	19.81	18.68	19.04	19.35	22.69	21.24	19.84	21.23
2127/2/18	b.c.	NA	22.18	30.42	78.12	33.83	21.67	33.7	21.48	32.06	20.59	32.31	20.87	34.14	23.42	33.1	23.35
2127/3/18	b.c.	NA	22.13	24.06	77.96	27.76	21.75	27.26	21.75	26.29	20.61	26.3	21.06	28.35	23.18	27.9	23.89
2093/2/18	b.c.	NA	25.41	20.1	77.96	25.59	25.21	23.47	23.55	23.56	23.52	22.92	24.27	26.26	26.73	23.63	26.87
2075/14	b.c.	NA	26.3	30.6	77.8	26.75	27.19	25.66	28.51	24.56	24.06	23.22	24.66	27.5	28.6	24.62	28.18
2074/14	b.c.	NA	23.23	24.38	77.96	32.75	31.46	24.91	24.43	20.77	20.65	21.91	21.52	23.99	24.44	23.11	24.91
2186/18	b.c.	NA	27.4	20.61	78.25	23.97	27.99	22.28	27.83	21.56	27.48	21.23	29.14	22.57	28.58	21.87	29.38
2139/18	s.	NA	28.24	26.73	78.39	28.8	27.42	27.2	26.7	27.84	27.88	26.73	28.77	28.39	28.75	26.58	29.1
2143/18	b.c.	NA	23.29	25.32	78.39	27.24	25.32	25.11	22.99	25.23	22.58	24.3	22.78	25.59	22.85	24.66	23.43
1942/1/18	b.c.	NA	26.34	20.85	78.53	26.27	27.58	24.23	26.23	23.89	25.86	23.96	26.86	24.81	26.51	22.81	27.13
3132/18	b.c.	NA	23.09	19.08	78.4	21.7	19.79	21.52	20.99	21.17	19.23	21.71	20.13	22.39	19.58	21.98	19.54
3133/18	b.c.	NA	28.16	31.73	78/82.56	37.53	26.4	35.94	27.02	35.03	25.37	35.47	25.35	38.54	27.15	35.8	25.13
2156/18	b.c.	NA	26.12	19.93	78.09	24.76	24.19	18.39	23.87	24.38	23.25	22.67	23.88	25.22	23.94	23.16	24.52
2167/18	b.c.	NA	27.16	19.76	78.09	23.32	26.17	17.7	23.82	21.52	23.52	20.8	24.16	22.97	25.01	22.1	25.81
2197/2/18	b.c.	NA	29.3	29.81	77.54	31.81	29.31	30.17	28.74	31.75	28.87	30.96	29.88	31.83	26.94	30.31	25.55

2188/2/18	b.c.	NA	28.44	31.38	77.94	31.61	26.9	29.2	27.29	32.51	27.97	30.46	28.26	31.71	24.79	29.93	25.29
2198/18	b.c.	NA	30.54	28.73	78.24	30.11	31.91	25.37	29.86	30.01	31.14	28.81	31.09	30.12	29.79	28.55	28.37
2129/18	b.c.	NA	25.18	17.79	78.27	24.83	26.0	23.59	26.58	23.88	24.82	22.96	24.55	24.37	24.88	23.57	25.13
2132/1/18	b.c.	NA	17.02	19.33	78.27	24.4	22.65	23.8	22.84	23.13	21.14	23.36	21.42	25.17	22.47	24.41	21.92
2155/18	b.c.	NA	24.84	18.45	78.41	24.16	24.62	22.15	25.1	24.49	25.77	23.35	25.95	24.73	25.13	22.39	22.66
2207/18	b.c.	NA	25.09	20.86	78.56	23.95	25.75	22.71	26.11	24.41	26.38	23.6	26.71	24.07	24.14	23.33	21.6
2208/18	b.c.	NA	23.17	20.25	78.26	21.91	22.08	22.81	23.35	23.83	24.76	22.46	25.32	24.05	21.71	22.58	23.42
2209/18	b.c.	NA	20.82	17.15	78.26	20.45	22.51	20.14	23.55	19.9	22.24	19.46	23.09	20.44	19.31	19.22	20.38
2220/18	b.c.	NA	24.16	24.78	78.43	26.64	24.26	26.15	23.9	28.48	24.51	26.1	24.1	28.86	23.13	26.68	23.01
2228/1/18	b.c.	NA	26.02	20.41	78.43	23.74	25.8	22.39	26.68	22.72	25.75	20.67	23.97	24.86	26.26	23.07	26.11
2228/2/18	b.c.	NA	27.69	20.5	78.23	24.3	27.24	22.38	28.29	22.92	26.21	20.57	25.0	24.94	30.38	22.94	28.49
2245/18	b.c.	NA	24.3	17.41	78.27	20.18	24.22	18.58	25.14	19.51	23.49	18.63	24.19	21.04	24.68	18.96	24.92
2243/18	b.c.	NA	23.37	20.23	77.96	21.94	23.84	21.94	23.18	21.07	23.26	19.75	23.12	22.37	24.18	21.15	23.9
1966/4/18	b.c.	NA	28.2	28.24	78.41	30.0	25.14	30.0	27.97	31.93	26.31	30.39	26.29	32.95	27.46	31.72	27.21
2497/14	b.c.	NA	31.91	33.36	78/82.46	29.9	26.79	29.9	31.63	36.13	30.92	34.08	30.47	36.41	32.51	34.45	31.68
1609/15	b.c.	NA	30.77	29.48	78.09	32.81	27.56	26.56	23.97	32.64	27.55	30.92	27.9	34.19	29.39	31.35	29.22
1614/15	b.c.	NA	30.1	23.93	78.09	27.38	26.81	25.47	26.08	25.59	26.27	24.98	26.24	28.54	28.26	26.09	29.32
1635/15	b.c.	NA	27.68	28.85	78.38	30.53	25.78	29.02	24.77	28.72	23.53	28.3	23.86	31.43	25.89	30.46	26.48
3442/21	w.b.	NA	21.45	14.84	78.38	18.2	21.39	17.66	22.73	16.68	21.59	15.85	22.28	17.92	21.36	16.19	22.44
3405/2/21	w.b.	NA	22.29	17.34	77.94	22.55	24.16	20.62	23.14	19.57	22.3	18.82	23.18	20.23	22.42	19.35	23.22
3050/2/21	w.b.	NA	16.89	16.91	78.24	20.78	20.75	20.35	22.34	19.66	19.08	18.46	19.56	21.14	20.45	19.36	21.31
3427/1/21	w.b.	NA	21.94	21.68	78.25	24.37	17.89	23.94	19.33	24.16	20.48	22.36	19.92	24.46	20.78	24.81	22.16
3427/2/21	w.b.	NA	22.26	22.99	78.25	27.29	21.55	24.54	21.35	25.53	21.2	24.6	21.16	26.0	21.06	25.48	21.6
3427/3/21	w.b.	NA	21.44	25.0	78.39	28.82	21.31	28.11	21.42	27.32	19.69	26.6	20.4	28.23	19.85	26.76	20.63
3433/1/21	w.b.	NA	21.84	16.6	78.09	20.91	21.16	18.74	20.79	NT	NT	NT	NT	20.68	19.94	19.95	21.77
3435/21	w.b.	NA	21.61	21.81	77.64	25.47	20.68	23.38	20.34	25.46	20.75	23.97	21.49	25.77	18.77	24.12	20.61
3436/21	w.b.	NA	25.43	28.55	78.09	31.87	23.89	28.6	23.29	32.36	24.16	31.16	24.63	32.69	23.43	31.69	24.21
3438/21	w.b.	NA	20.37	15.66	78.1	20.64	23.87	17.97	22.74	20.07	24.07	18.53	24.27	19.17	21.32	17.78	22.71
3440/21	w.b.	NA	21.58	18.5	78.13	21.74	21.81	18.06	20.19	21.53	22.34	18.86	21.87	21.64	20.6	19.79	21.08
1381/23	w.b.	34.76	19.76	31.41	79.16	34.35	19.42	33.18	20.02	32.95	17.55	33.36	18.9	36.82	18.67	34.17	19.66
1449/23	w.b.	24.68	23.3	18.21	79.62	19.75	22.78	17.87	22.72	18.49	21.57	16.31	22.35	18.69	22.65	17.46	23.47
1451/23	w.b.	24.55	22.49	17.8	79.33	18.8	22.13	18.18	22.07	18.42	21.78	17.37	22.69	20.17	23.24	17.43	22.93
1452/23	w.b.	27.18	21.27	22.28	79.33	22.93	20.63	20.74	20.55	21.52	20.23	19.87	20.69	21.73	20.97	20.78	21.51
1466/23	w.b.	23.52	19.62	18.68	79.29	17.97	19.03	17.03	19.55	18.47	18.9	15.33	19.37	20.09	20.54	16.93	20.26
2032/23	serum	32.54	30.31	25.04	79.44	28.65	30.39	25.78	29.74	27.31	29.92	23.39	29.37	26.7	29.03	25.52	30.82
1408/23	w.b.	22.4	21.58	22.69	79.42	17.66	22.07	16.42	22.88	17.05	21.67	15.34	22.29	18.15	22.46	15.36	23.76
1417/23	w.b.	30.8	18.31	27.16	79.57	18.18	16.55	17.25	18.35	17.71	16.97	15.48	17.43	18.99	17.78	16.84	18.33

1419/1/23	w.b.	32.19	20.66	31.41	79.27	26.49	20.03	25.95	21.32	25.87	19.62	23.44	20.67	26.54	20.72	27.48	22.27
1419/2/23	w.b.	23.23	20.01	21.94	79.43	17.5	18.34	17.18	20.08	17.25	18.57	15.38	19.2	20.03	20.62	15.55	19.93
1419/3/23	w.b.	21.91	21.3	21.83	79.43	18.7	21.3	16.85	21.8	17.43	19.61	14.64	19.75	17.8	21.45	16.27	20.93
1458/23	w.b.	32.21	21.47	23.87	79.57	20.35	20.57	18.48	21.45	20.09	20.37	17.76	20.65	19.29	21.33	17.97	21.97
1469/1/23	w.b.	22.66	19.17	22.37	79.57	19.83	19.01	17.41	19.65	18.35	17.52	16.73	18.13	19.18	18.75	16.67	18.97
1476/1/23	w.b.	26.14	21.23	26.2	78.98	23.02	20.76	20.37	21.4	21.81	20.11	20.27	20.34	22.11	19.13	20.39	20.51
1476/2/23	w.b.	31.67	23.08	31.91	79.43	27.67	22.61	26.6	22.71	26.56	21.33	25.49	22.16	26.25	20.11	25.27	20.64
1476/3/23	w.b.	25.57	18.76	21.99	79.65	23.68	20.75	21.79	20.47	21.31	18.35	19.93	19.01	22.1	17.44	20.76	18.68
1478/1/23	w.b.	27.91	19.44	30.62	79.28	25.57	19.56	23.26	19.09	17.96	22.93	22.21	18.35	23.77	17.24	22.68	17.58
1478/4/23	w.b.	24.71	20.66	26.86	79.27	22.67	20.34	19.15	19.75	21.62	19.96	18.98	19.94	21.95	18.06	19.94	19.35
1478/5/23	w.b.	27.79	21.73	22.4	79.79	24.65	21.13	22.39	20.49	22.93	19.22	21.41	19.51	23.89	18.6	21.59	18.31
1477/23	w.b.	20.87	20.32	15.57	79.64	14.5	19.73	14.98	18.71	15.36	18.94	14.12	19.89	18.37	19.98	14.83	20.82
1480/1/23	w.b.	22.1	20.35	15.36	79.33	18.53	21.29	15.91	20.77	15.95	19.51	14.14	20.59	17.24	19.6	17.82	20.52
1480/3/23	w.b.	21.01	18.29	19.7	79.64	19.12	17.86	15.63	18.17	17.98	16.4	16.42	17.04	19.3	17.78	16.71	17.59
1481/23	w.b.	21.3	20.06	17.07	79.5	18.29	19.64	15.74	20.42	16.19	17.76	14.66	18.89	17.14	18.13	15.45	19.31
1482/3/23	w.b.	28.77	21.39	17.84	79.2	17.45	21.31	12.84	21.14	16.22	20.55	14.59	20.56	18.06	20.64	15.2	21.73
1482/5/23	w.b.	NA	22.45	30.89	79.79	34.33	21.96	32.9	22.18	32.74	20.95	32.16	20.69	34.78	22.73	29.83	23.23
1968/23	serum	32.05	30.88	24.99	79.5	27.67	31.96	24.84	31.41	26.64	30.5	25.2	31.08	26.99	29.32	24.3	30.28
1969/23	serum	31.92	32.17	27.98	79.45	28.38	31.37	27.31	33.14	27.87	31.36	26.34	32.49	27.87	30.72	25.99	31.98
vac K-KB	in. vac	40.37	NA	25.13	77.77	21.49	39.19	23.72	42.28	23.86	NA	19.61	NA	23.8	40.88	21.15	37.71
**1361/24**	**w.b.**	**NA**	**22.43**	**34.86**	**79.29**	**NA**	**20.64**	**NA**	**21.19**	**NA**	**21.09**	**NA**	**21.01**	**NA**	**19.38**	**NA**	**21.25**
**1362/24**	**s.**	**NA**	**24.55**	**35.81**	**79.74**	**NA**	**19.26**	**NA**	**21.98**	**NA**	**22.16**	**NA**	**22.6**	**NA**	**22.07**	**NA**	**23.17**
**1299/4/24**	**w.b.**	**NA**	**18.67**	**36.76**	**78.12**	**NA**	**18.16**	**NA**	**18.83**	**NA**	**16.76**	**NA**	**16.99**	**NA**	**19.15**	**NA**	**19.66**

Stram- assay designed by Stram et al. [21]; Erster- assay designed by Erster et al.[17]; Sensi- SensiFAST SYBR® No-ROX Kit (Bioline) was used for Erster et al. assay [21]; all the last- comparative tests of Mix-2 and Mix-8 recently validated assays; Ct- cycle threshold; w.b.- whole blood; b.c.- buffy coat; P.- plasma; s.- spleen; s+l- mix of spleen and lung; NA- not amplified; b -ACT- ß- actin; Quanta - qScript XLT One-Step RT-qPCR ToughMix(Quantabio); AgPath- AgPath-ID™ One-Step RT-PCR Kit (Life technologies); Clara- Clara™ Probe 1-Step Mix No-ROX (PCR biosystems); Promega- GoTaq 1-Step RT-qPCR System (Promega); Takara- One Step PrimeScript™ RT-PCR Kit (Takara Bio Inc.); Tm- melting temperature. The horizontal line separates negative from positive samples. Samples signed in bold, showed different results (positive vise negative, and vise versa) in different RT-qPCR assays
